# Case report: *EGFR* fusion mutation combined with *EGFR* amplification responds to EGFR-TKI therapy

**DOI:** 10.3389/fonc.2024.1347282

**Published:** 2024-03-25

**Authors:** Zhulin Wang, Chunyao Huang, Wenbo Fan, Shaowu Sun, Kaiyuan Li, Xu Liu, Jiangtao Pu, Guoqing Zhang, Xiangnan Li

**Affiliations:** ^1^ Department of Thoracic Surgery, Affiliated Hospital of Southwest Medical University, Luzhou, Sichuan, China; ^2^ Department of Thoracic Surgery, First Affiliated Hospital of Zhengzhou University, Zhengzhou, Henan, China

**Keywords:** EGFR fusion, EGFR amplification, rare mutations, lung adenocarcinoma, targeted therapy

## Abstract

Given their good antitumor effects, epidermal growth factor receptor (*EGFR*) tyrosine kinase inhibitors (TKIs) are standard first-line therapy for *EGFR*-sensitive mutations, including exon 19 deletions and exon 21 L858R mutations. *EGFR* fusion mutations and *EGFR* amplification are very rare in non-small cell lung cancer (NSCLC). We describe 2 patients with NSCLC harboring *EGFR* fusion mutations (*EGFR-MACF1* and *EGFR-GNAT3*) combined with *EGFR* amplification. Both patients received EGFR-TKI treatment, and 1 of them showed an antitumor response.

## Introduction

Lung cancer is one of the leading causes of cancer-related death worldwide (1), and EGFR-TKIs are the standard first-line treatment for patients with NSCLC with sensitive *EGFR* mutations (2). *EGFR* gene fusion mutations are rare, and currently reported *EGFR* fusion mutations include *EGFR-RAD51, EGFR-PURB, EGFR-ANXA2, EGFR-IGR*, etc. *EGFR* gene fusion mutations combined with *EGFR* amplification are even rarer. Therefore, the optimal treatment for lung cancer patients with *EGFR* fusion mutations and *EGFR* amplification is unclear. We previously reported a patient (3) with an *EGFR* fusion mutation (*EGFR-IGR*) with *EGFR* amplification. After 2 months of treatment with gefitinib and cetuximab, the tumor shrank significantly, followed by right upper lobectomy and mediastinal lymph node dissection. The patient’s last follow-up was on March 4, 2023, with an OS > 30 months (**Supplementary Figure 1**). Previous preclinical and cell studies have shown that NSCLC patients with *EGFR* fusion mutations benefit from EGFR-TKI treatment (4, 5). In addition, patients with *EGFR*-sensitive mutations combined with *EGFR* amplification seem to have better antitumor responses to treatment with EGFR-TKIs (6). Therefore, we try to treat patients with *EGFR* fusion mutations combined with *EGFR* amplification with EGFR-TKI therapy. We describe two patients with *EGFR* fusion mutations (*EGFR-MACF1* and *EGFR-GNAT3*) combined with *EGFR* amplification and provide detailed information, including the gene fusion location and response to TKI therapy.

## Case description

**Figure 1:**
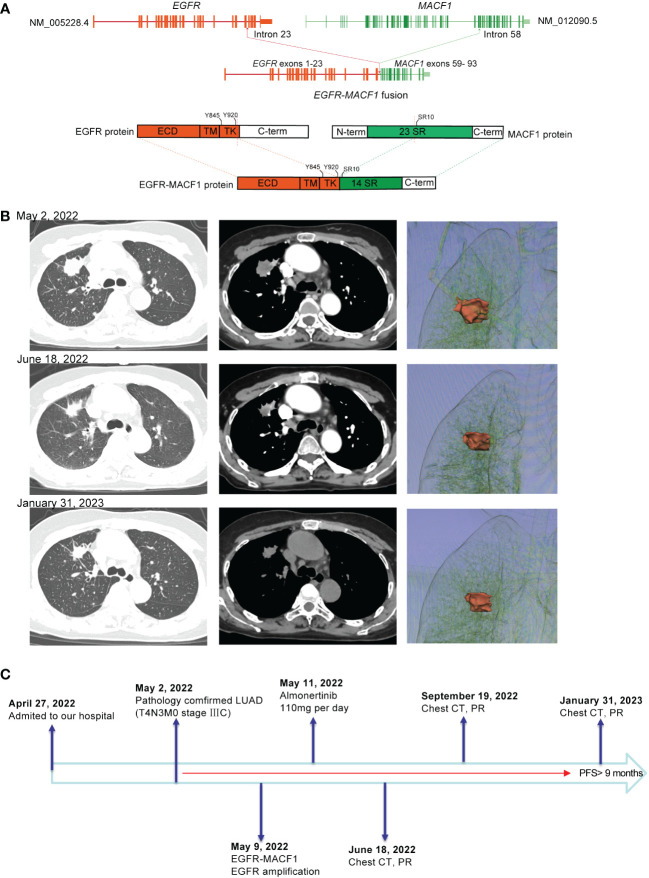
**(A)** Schematic diagram of the domain structure of the EGFR-MACF1 fusion at the RNA and protein levels. **(B)** Computed tomography (CT) scan and three-dimensional reconstruction before and after EGFR-TKI treatment and at the latest follow-up. **(C)** The entire treatment procedure.

Patient 1, a 65-year-old female, was admitted to the hospital due to chest pain and shoulder and back pain on April 27, 2022. A chest computed tomography (CT) scan showed a 2.4 cm mass in the upper lobe of the right lung, multiple right lung metastases, and mediastinal lymph node metastasis. CT-guided biopsy of the right lung lesion revealed that the mass was lung adenocarcinoma, and the patient was subsequently diagnosed with lung adenocarcinoma (T4N3M0 stage IIIC, AJCC8TH). On May 9, 2022, 86 cancer-related genes were detected in tissue samples by next-generation sequencing (NGS). The *EGFR* gene was fused with the *MACF1* gene at the RNA level (mutation abundance: 17%), and *EGFR* was amplified (copy number: 24.15). The *EGFR-MACF1* gene included *EGFR* exons 1–23 and *MACF1* exons 59–93 (**Figure 1A**). After discussing the patient’s condition, the Lung Cancer Multidisciplinary Team (MDT) recommended treatment with almonertinib (110 mg/day) on May 11, 2022. After 1 month of treatment, a chest CT showed significant shrinkage of the mass in the patient’s right upper lobe. Afterward, the patient was re-examined every 3 months. Re-examination by chest CT on January 31, 2023, revealed that the tumor continued to respond to the EGFR-TKI (**Figure 1B**). According to the RECIST guidelines, the patient was considered to have a partial response to almonertinib, and the patient’s progression-free survival (PFS) was >9 months (**Figure 1C**).

**Figure 2:**
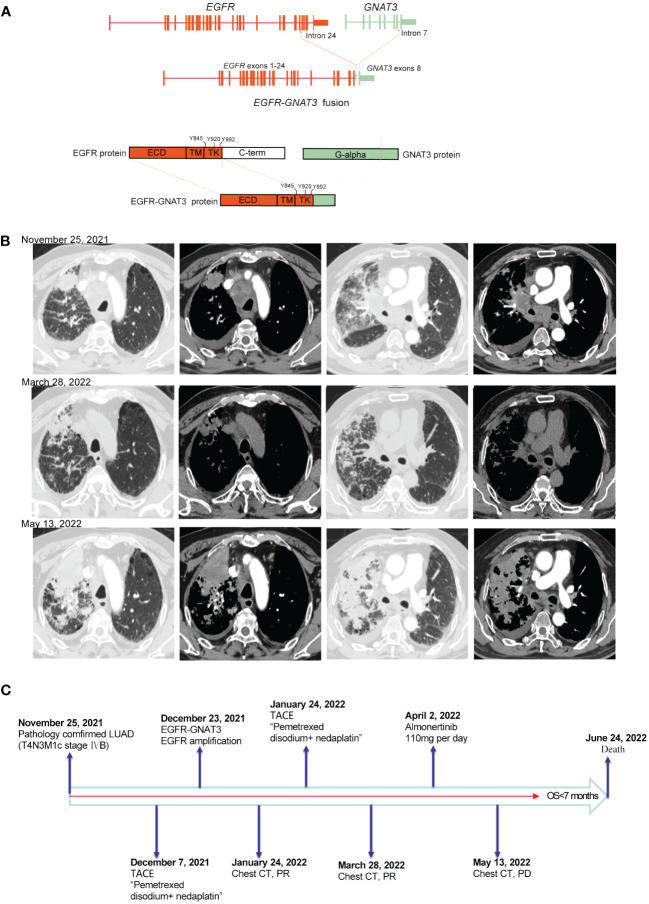
**(A)** Schematic diagram of the domain structure of the EGFR-GNAT3 fusion at the DNA and protein levels. **(B)** Computed tomography (CT) scan before and after treatment and after tumor progression. **(C)** The entire treatment procedure.

Patient 2, a 58-year-old male, experienced cough, sputum, chest tightness, and back pain on December 3, 2021. Chest CT and positron emission tomography (PET)-CT scans revealed masses in the right upper lobe and right hilum of the patient, with the larger mass measuring 3.2 cm, multiple intrapulmonary metastases, multiple lymph node metastases throughout the body (including the mediastinum, hilar, bilateral neck, bilateral clavicle region, left armpit, etc.) and multiple bone metastases (right 7th rib, left 9th rib, 9th thoracic vertebra, etc.). CT-guided biopsy of the right lung lesion revealed that the mass was lung adenocarcinoma, which was diagnosed as lung adenocarcinoma (T4N3M1c stage IVB, AJCC8TH). On December 23, 2021, 14 cancer-related genes were detected in tissue samples using NGS. *EGFR* gene fusion with the *GNAT3* gene (mutation abundance: 76.3%) and *EGFR* amplification (copy number: 8.1). The *EGFR-GNAT3* gene included *EGFR* exons 1–24 and *GNAT3* exon 8 (**Figure 2A**). The patient initially underwent arterial chemoembolization (protocol: pemetrexed disodium and nedaplatin), which resulted in mass shrinkage. However, due to physical reasons specific to the patient, the drug was suspended for 2 months, after which the tumor progressed after 1 month of treatment with almonertinib (110 mg/day) (**Figure 2B**). On June 24, 2022, the patient died of severe lung infection and systemic multiple organ failure, with an overall survival (OS) <7 months (**Figure 2C**). Additional information regarding the 2 patients is summarized in **Table 1**.

**Table 1:** Overview of the 2 patients

Pt NO.	Age	Gender	Diagnosis	Stage	Mutations	EGFR-TKI Treatment	Response to TKI	PFS (months)	OS (months)
1	65	Female	LUAD	IIIC	*EGFR- MACF1* and *EGFR* amp	Almonertinib	PR	>9	>9
2	58	Male	LUAD	IVB	*EGFR-GNAT3* and *EGFR* amp	Almonertinib	PD	1.3	<7

LUAD, Lung adenocarcinoma; PR, Partial response; PD, Progressive disease; PFS, progression-free survival; OS, Overall survival.

## Discussion

*EGFR* activation is a dimerization reaction that results in a transformation from an inactive to an active conformation as the local concentration of the receptor increases (7). *EGFR* activation occurs due to the formation of an asymmetric dimer (7). In addition, *EGFR* contains several autophosphorylation sites in the C-terminal tail of the receptor (including tyrosines 992, 1068, and 1173) (8, 9). Dimerization leads to phosphorylation of tyrosine residues in the C-terminal tail, which in turn activates the PI3K/AKT and MAPK oncogenic pathways.

In the model constructed by Kartik et al. (10), the *EGFR-RAD51* fusion protein was shown to contribute an oligomerization domain through RAD51 to promote kinase activation. However, among other fusion partners, such as *EGFR-IGR, EGFR-ANXA2, EGFR-SEPTIN14*, and *EGFR-SHC1*, no studies have shown that the fusion partners involve oligomerization domains. In addition, upon *EGFR* fusion, a subset of phosphorylation sites critical for intact *EGFR* function and transformation may be preserved, and these phosphorylation sites may be oncogenic (8). In our study, although the fusion sites of the three patients were different, they all retained phosphorylation sites that may cause cancer (patient 1: tyrosine 845, tyrosine 920; patient 2: tyrosine 845, tyrosine 920, tyrosine 992; patient 3: tyrosine 845, tyrosine 920, tyrosine 992).

Previous studies have shown that patients with tumors harboring *EGFR* fusions can benefit clinically from EGFR-TKI therapy (3, 11, 12). In cell experiments, EGFR-TKIs inhibited the growth of BA/F3 cells harboring the *EGFR* fusion protein to varying degrees (10).

According to the case series in this study, we found that *EGFR* gene fusions are often accompanied by *EGFR* amplification. The results of Shigenari et al (13) show that the amplification of EGFR wild-type (rather than mutant EGFR) alleles may induce acquired drug resistance to third-generation EGFR-TKIs through activation induced by EGFR ligands. We recently reported on the combined targeted therapy-”sandwich” regimen (14). This strategy was successfully applied in a patient with *EGFR-IGR* combined with *EGFR* amplification (3). The fundamental principle involves using *EGFR* monoclonal antibodies to target EGFR amplification and EGFR-TKIs to target EGFR fusion. However, there is evidence that primary *EGFR* amplification may be effective for EGFR-TKI targeted therapy. Ruiz-Patiño et al. (15) and Shan et al. (6) found that patients with *EGFR* mutations and *EGFR* amplification exhibited significant antitumor responses when treated with EGFR-TKIs and had better survival than patients without amplification. Furthermore, treatment with the first-generation TKI larotrectinib resulted in significant antitumor activity in patients with advanced ESCC with *EGFR* overexpression or amplification (16). However, some previous studies have shown that *EGFR* amplification in untreated patients after TKI treatment may lead to drug resistance in patients receiving TKIs. Nitin et al (17) reported 5 patients (35.7%) had EGFR amplification in patients with drug resistance after treatment with oxitinib. These results suggest that *EGFR* amplification in untreated patients after TKI treatment may lead to drug resistance to TKIs. In the study of Helman et al (18), 17 of the 58 patients who progressed when they were treated with rociletinib had *EGFR* amplification. Taken together, these findings indicate that primary *EGFR* amplification may be effective for TKI therapy, while secondary *EGFR* amplification mediates TKI resistance. Therefore, discussions among the Lung Cancer Multidisciplinary Team (MDT) resulted in the recommendation for the use of single-drug TKI therapy in patients with rare *EGFR* fusion mutations and *EGFR* amplification. The data presented in these case studies were obtained with informed consent from each patient, and the study was approved by the Zhengzhou University Institutional Review Board.

Patient 1 achieved good clinical efficacy. Patient 2 was under the care of another medical team and was discovered when reviewing cases for this study; this was a negative case. The patient inappropriately received local intervention as a first-line therapy without systemic therapy, which led to progression of the systemic disease and a decline in the patient’s physical condition. Although a TKI was chosen for treatment in the later stage, the optimal time for treatment was missed. After the patient received targeted therapy for one month, the results showed that it was ineffective. These findings also show the importance of early systemic treatment for patients with advanced lung cancer. Additionally, we do not know whether new mutations that appeared after the previous treatment led to the poor efficacy of EGFR-TKI therapy observed in this patient because additional NGS testing was not performed after the disease had progressed.

## Conclusions

Here, we present 2 patients with NSCLC with *EGFR* fusions combined with *EGFR* amplification, both of whom represented rare cases. One of the patients showed a significant antitumor response after EGFR-TKI treatment. Future studies should involve basic research on these rare mutations to explore their cancer-causing mechanisms.

## Data availability statement

The original contributions presented in the study are included in the article/**Supplementary Material**. Further inquiries can be directed to the corresponding authors.

## Ethics statement

The studies involving humans were approved by the Ethics Committee of Zhengzhou University School of Medicine. The studies were conducted in accordance with the local legislation and institutional requirements. The participants provided their written informed consent to participate in this study. Written informed consent was obtained from the individual(s) for the publication of any potentially identifiable images or data included in this article. We confirm that written informed consent has been obtained from the participant/patient(s) for the publication of this case report. Written informed consent was obtained from the participant/patient(s) for the publication of this case report.

## Author contributions

ZW: Conceptualization, Data curation, Formal analysis, Investigation, Methodology, Writing – original draft, Writing – review & editing. CH: Formal analysis, Methodology, Project administration, Writing – original draft. WF: Data curation, Formal analysis, Methodology, Project administration, Writing – review & editing. SS: Formal analysis, Investigation, Project administration, Writing – original draft. KL: Data curation, Formal analysis, Investigation, Writing – review & editing. XL: Conceptualization, Formal analysis, Writing – review & editing. JP: Resources, Writing – review & editing, Conceptualization, Data curation. GZ: Resources, Writing – review & editing, Formal analysis, Supervision. XNL: Funding acquisition, Methodology, Resources, Writing – review & editing.

## Supplementary material

Supplementary Figure 1 A Schematic diagram of the domain structure of the EGFR-IGR fusion at the DNA and protein levels. ECD: extracellular domain, TM: transmembrane domain, TK: tyrosine kinase domain; Y845: tyrosine 845, Y920: tyrosine 920, Y992: tyrosine 992. 1B, The entire treatment procedure.

## References

[1] Siegel RL Miller KD Wagle NS Jemal A. Cancer statistics, 2023. CA Cancer J Clin. (2023) 73:17–48. doi: 10.3322/caac.21763 36633525

[2] He J Huang Z Han L Gong Y Xie C. Mechanisms and management of 3rd−generation EGFR−TKI resistance in advanced non−small cell lung cancer (Review). Int J Oncol. (2021) 59. doi: 10.3892/ijo.2021.5270 PMC8562388 34558640

[3] Zhang G Xia P Zhao S Yuan L Wang X Li X. Gefitinib combined with cetuximab for the treatment of lung adenocarcinoma harboring the EGFR-intergenic region (SEC61G) fusion and EGFR amplification. Oncologist. (2021) 26:e1898–902. doi: 10.1002/onco.13921 PMC8571749 34342091

[4] Copia Sperandio R Luiza Teixeira Tostes F Vidal Campregher P Ribeiro Paes V Moura F Schvartsman G. EGFR-RAD51 fusion in lung adenocarcinoma with systemic and intracranial response to osimertinib: A case report and review of the literature. Lung Cancer. (2022) 166:94–7. doi: 10.1016/j.lungcan.2022.02.006 35245845

[5] Guan Y Song Z Li Y Guo H Shi J Zhang X. Effectiveness of EGFR-TKIs in a patient with lung adenocarcinoma harboring an EGFR-RAD51 fusion. Oncologist. (2019) 24:1027–30. doi: 10.1634/theoncologist.2018-0732 PMC6693705 31064887

[6] Shan L Wang Z Guo L Sun H Qiu T Ling Y. Concurrence of EGFR amplification and sensitizing mutations indicate a better survival benefit from EGFR-TKI therapy in lung adenocarcinoma patients. Lung Cancer. (2015) 89:337–42. doi: 10.1016/j.lungcan.2015.06.008 26141217

[7] Zhang X Gureasko J Shen K Cole PA Kuriyan J. An allosteric mechanism for activation of the kinase domain of epidermal growth factor receptor. Cell. (2006) 125:1137–49. doi: 10.1016/j.cell.2006.05.013 16777603

[8] Jorissen RN Walker F Pouliot N Garrett TP Ward CW Burgess AW. Epidermal growth factor receptor: mechanisms of activation and signalling. Exp Cell Res. (2003) 284:31–53. doi: 10.1016/s0014-4827(02)00098-8 12648464

[9] Kwon Y Kim M Jung HS Kim Y Jeoung D. Targeting autophagy for overcoming resistance to anti-EGFR treatments. Cancers (Basel). (2019) 11. doi: 10.3390/cancers11091374 PMC6769649 31527477

[10] Konduri K Gallant JN Chae YK Giles FJ Gitlitz BJ Gowen K. EGFR fusions as novel therapeutic targets in lung cancer. Cancer Discovery. (2016) 6:601–11. doi: 10.1158/2159-8290.Cd-16-0075 PMC4893907 27102076

[11] Zhu YC Wang WX Xu CW Song ZB Du KQ Chen G. EGFR-RAD51 fusion variant in lung adenocarcinoma and response to erlotinib: A case report. Lung Cancer. (2018) 115:131–4. doi: 10.1016/j.lungcan.2017.12.001 29290255

[12] Di Federico A Filetti M Palladini A Giusti R Piras M De Giglio A. EGFR-RAD51 gene fusion NSCLC responsiveness to different generation EGFR-TKIs: two cases and review of the literature. Transl Lung Cancer Res. (2022) 11:497–503. doi: 10.21037/tlcr-21-888 35399574 PMC8988076

[13] Nukaga S Yasuda H Tsuchihara K Hamamoto J Masuzawa K Kawada I. Amplification of EGFR wild-type alleles in non-small cell lung cancer cells confers acquired resistance to mutation-selective EGFR tyrosine kinase inhibitors. Cancer Res. (2017) 77:2078–89. doi: 10.1158/0008-5472.Can-16-2359 28202511

[14] Zhang G Yan B Guo Y Yang H Li J. "Sandwich" Strategy to intensify EGFR blockade by concurrent tyrosine kinase inhibitor and monoclonal antibody treatment in highly selected patients. Front Oncol. (2022) 12:952939. doi: 10.3389/fonc.2022.952939 35903676 PMC9321780

[15] Ruiz-Patiño A Castro CD Ricaurte LM Cardona AF Rojas L Zatarain-Barrón ZL. EGFR amplification and sensitizing mutations correlate with survival in lung adenocarcinoma patients treated with erlotinib (MutP-CLICaP). Target Oncol. (2018) 13:621–9. doi: 10.1007/s11523-018-0594-x 30284706

[16] Liu R Liu L Zhao C Bai Y Zheng Y Zhang S. Larotinib in patients with advanced and previously treated esophageal squamous cell carcinoma with epidermal growth factor receptor overexpression or amplification: an open-label, multicenter phase 1b study. BMC Gastroenterol. (2021) 21:398. doi: 10.1186/s12876-021-01982-4 34688250 PMC8540164

[17] Roper N Brown AL Wei JS Pack S Trindade C Kim C. Clonal evolution and heterogeneity of osimertinib acquired resistance mechanisms in EGFR mutant lung cancer. Cell Rep Med. (2020) 1. doi: 10.1016/j.xcrm.2020.100007 PMC7263628 32483558

[18] Helman E Nguyen M Karlovich CA Despain D Choquette AK Spira AI. Cell-free DNA next-generation sequencing prediction of response and resistance to third-generation EGFR inhibitor. Clin Lung Cancer. (2018) 19:518–30.e517. doi: 10.1016/j.cllc.2018.07.008 30279111

