# Supplementary figures and images for: Case report: EGFR fusion mutation combined with EGFR amplification responds to EGFR-TKI therapy
Source: Front Oncol. 2024 Mar 25;14:1347282. doi: 10.3389/fonc.2024.1347282 (PMC11003264; doi:10.3389/fonc.2024.1347282)

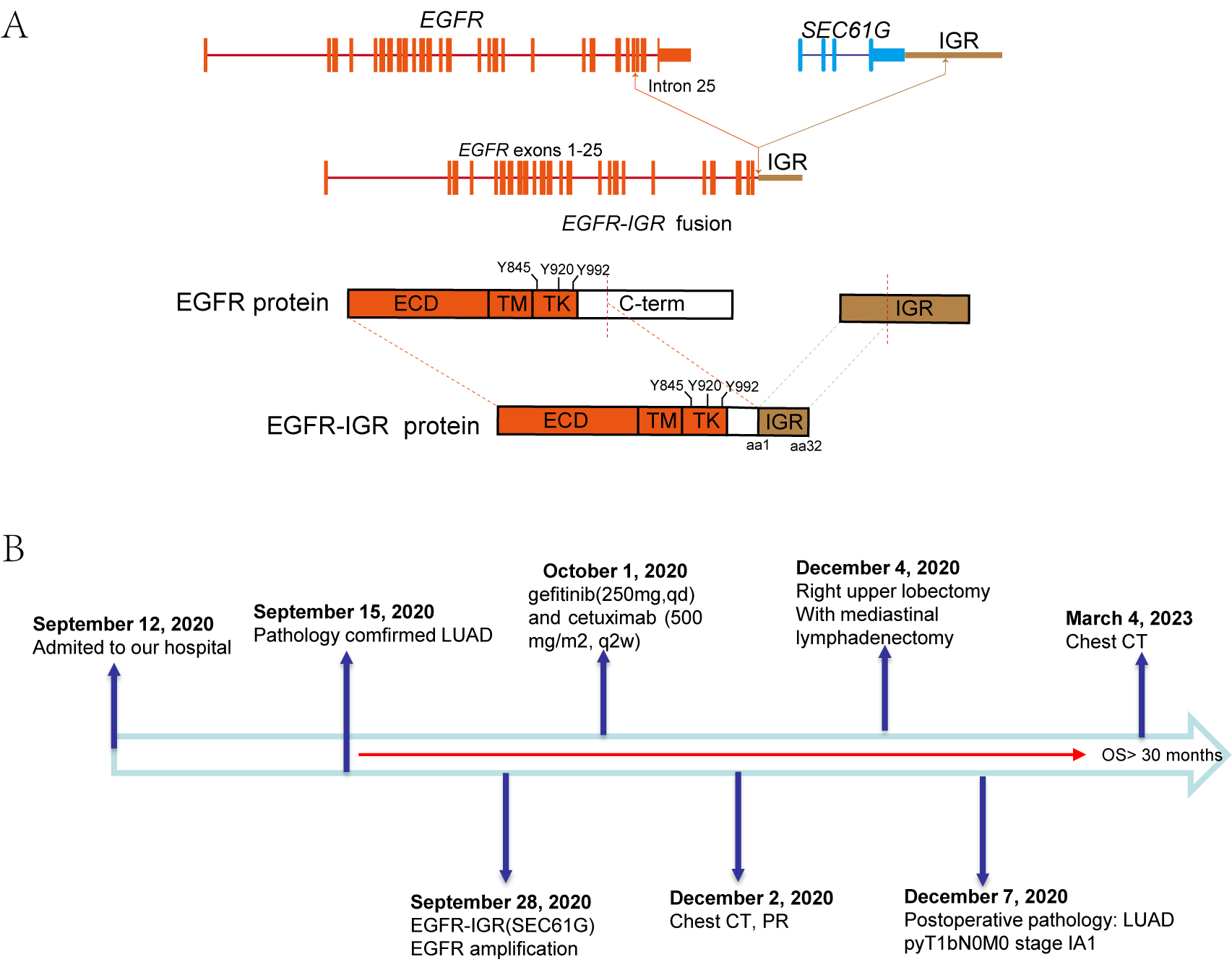

Supplement: Supplementary Figure 1 — A Schematic diagram of the domain structure of the EGFR-IGR fusion at the DNA and protein levels. ECD: extracellular domain, TM: transmembrane domain, TK: tyrosine kinase domain; Y845: tyrosine 845, Y920: tyrosine 920, Y992: tyrosine 992. 1B, The entire treatment procedure. [file Image_1.tif]
